# Editorial on the validity of plain radiographs in low-grade periprosthetic hip infections

**DOI:** 10.1080/07853890.2024.2352590

**Published:** 2024-06-04

**Authors:** Filippo Migliorini, Ulf Krister Hofmann

**Affiliations:** Department of Orthopaedic, Trauma, and Reconstructive Surgery, RWTH University Medical Centre, Aachen, Germany; Department of Orthopaedics and Trauma Surgery, Academic Hospital of Bolzano (SABES-ASDAA), Teaching Hospital of the Paracelsus Medical University, Bolzano, Italy

## Editorial

The diagnosis of low-grade periprosthetic joint infections (PJI) in total hip arthroplasty (THA) is challenging. Conventional radiography in symptomatic hip following arthroplasty should be undertaken. This methodology is mostly useful to exclude diagnoses such as fracture, third-body wear, heterotopic ossification, or component malpositioning. However, the reliability of conventional radiology in the diagnosis of low-grade PJI of the hip remains uncertain. Our understanding of the radiographic features in low-grade PJI in THA is only based on a few, weak, heterogeneous, and old evidence [1–14]. In aseptic loosening, radiographic changes are slow and progressive, while infectious loosening occurs rapidly, in a more aggressive manner and with greater bone destruction [14,15]. Claimed radiographic signs of infection are periosteal reaction, formation of lamellae, focal osteolysis or bone destruction, a wide radiolucent zone at the cement-bone or metal-bone interface, signs of loosening in a previously well-fixed implant, patchy osteolysis, heterotopic bone formation, mottling, intracortical sinus tract, periprosthetic fractures, adjacent soft tissue collection, scalloped endosteal bone resorption, and cement mantle irregularities. Aseptic loosening tends to produce uniform radiolucency, whereas particle disease produces multifocal radiolucencies related to localised osteolysis. Infection can produce either of these patterns, with osteolysis greater than 2 mm being considered pathognomonic.

Evidence on the specificity and sensitivity of these radiographic features in periprosthetic hip infections is inconsistent. Some authors report radiographic changes in all PJIs [16–18], other authors only found abnormalities in 14% to 63% [19–22]. Validity analyses reported a low sensitivity of 20-25% with a moderate to good specificity of periosteal reactions and periarticular calcifications (79 to 92%) [12,23] or scalloped bone resorption (96%) [12]. This evidence supports the role of plain radiographs in the diagnosis of low-grade PJI in THA. The reasons for these wide ranges of reported results are multifactorial. The studies considered included a small sample size (mostly 6 to 20) and a variable length of the follow-up ranging from days to decades, impairing reliable statistical estimates. Moreover, PJIs and their courses are influenced by various factors, including pathogen characteristics, patient comorbidities, or implant fixation techniques. Most important, however, are probably the lack of a standardised radiologic classification for PJIs and reliable time points chosen after surgery/infection for the evaluation of radiographic changes. The radiographic presentation of PJIs is a function of time, with early infections presenting no radiographic features. The first radiographic changes appear three months after the onset of the symptoms [17]. However, the evidence of radiographic presentation of PJIs is limited and requires additional investigations. The authors hypothesised that the validity of conventional radiology in PJIs within the first postoperative year may be inconsistent, being radiographic features of low-grade infections present not earlier than one year postoperatively. Future investigations should exclusively analyse patients with suspected low-grade infections after the second postoperative year.

The joint arthroplasty landscape has considerably changed over the past decades. THA is highly standardised, many implants are cementless, modern coating surfaces and ultra-high molecular weight (UHMW)-cross-linked vitamin-E-enriched polyethylene liners outperform their predecessors. The definition of PJIs and related diagnostic algorithms have also considerably changed, with improved and standardised bacteria culture, histopathological analyses, and the introduction of polymerase chain reaction assay [24–27]. Improves in digital radiography allow post-imaging optimisation to additionally visualise soft tissues: traditional radiology was optimised for bone contrast only.

Concluding, evidence on the radiologic features of low-grade PJIs in THA is lacking. High-quality analyses with larger sample sizes and a standardised approach concerning imaging time-point and image analysis criteria are required to establish the diagnostic validity of plain radiography in low-grade PJI. In that context, future investigations should also evaluate if there is an association between radiographic features and the underlying causative microorganism. Imaging (e.g. conventional radiography, computer tomography, and magnetic resonance) and nuclear medicine (e.g. scintigraphy, and positron emission tomography) are routinely used to diagnose PJI. Several clinical, serum, and synovial tests and microbiologic and histologic examinations assist in yielding a diagnosis. However, the reliability of these methodologies in the diagnosis of PJI still remains debated.

## Data Availability

No new data has been generated in the current study.
